# Current relevance of biomarkers in diagnosis of periprosthetic joint infection: an update

**DOI:** 10.1186/s42836-023-00192-5

**Published:** 2023-08-01

**Authors:** Saksham Tripathi, Saad Tarabichi, Javad Parvizi, Ashok Rajgopal

**Affiliations:** 1 Institute of Musculoskeletal Disorders and Orthopaedics, Medanta-The Medicity, Gurugram, HR 122001 India; 2 Rothman Orthopaedic Institute at Thomas Jefferson University Hospital, Philadelphia, PA 19107 USA

**Keywords:** Periprosthetic joint infection, Biomarkers, Diagnosis, Serum, Synovial

## Abstract

With a significant rise in the number of arthroplasty procedures performed worldwide, the increasing revision burden posed by periprosthetic joint infection (PJI) is a matter of growing concern. In spite of various attempts to diagnose PJI, there are no defined tests that can be called a gold standard. Given the importance of early diagnosis in PJI, newer tests and biomarkers have been introduced to improve cumulative diagnostic accuracy. Novel biomarkers like calprotectin, lipocalcin, monocyte-to-lymphocyte ratio, neutrophil-to-lymphocyte ratio, platelet-to-lymphocyte ratio and platelet-to-mean platelet volume ratio have demonstrated a potential as diagnostic biomarkers for PJI. This article discusses the relevance of available and newly described diagnostic biomarkers to provide a perspective on the practical applicability in current medical practice, as well as highlights some recent advances in biomarkers for the diagnosis of PJI.

## Introduction

An increase in life expectancy and demand for improved quality of life has resulted in a rise in the number of total joint arthroplasty (TJA) procedures performed globally [1, 2]. At the same time, improvements in perioperative care have led to substantial reduction in the risk of infection. The European Centre for Disease Prevention and Control’s (ECDC) surgical site infection (SSI) surveillance network reported an infection rate of 0.5% for patients undergoing knee replacement and a rate of 1% for those undergoing total hip replacement, with considerable variation in rates between countries [3]. In the United States, the cumulative incidences of infection were 0.5%, 0.8%, and 1.4% at 1, 5, and 10 years, respectively, after primary TJA [4].

PJI is the second commonest cause of revision total knee arthroplasty and the third leading cause for revision total hip arthroplasty [5–7].

PJI was the underlying reason in 30.5% revision total knee arthroplasties and 12% revision total hip arthroplasties as reported by the Indian Joint Registry [8].

In spite of various attempts to diagnose PJI, there are no defined tests that can be called a gold standard. Given the importance for early diagnosis in PJI, newer tests and biomarkers have been introduced to improve the cumulative diagnostic accuracy.

Erythrocyte sedimentation rate (ESR), serum C-reactive protein (CRP), synovial fluid cell counts and leukocyte esterase are included in the ICM criteria. Fibrinogen, procalcitonin, and D dimers have been used as additional tools for better accuracy. Novel biomarkers, calprotectin, soluble Pe-Cam 1, lipocalcin, D-lactate, monocyte-to-lymphocyte ratio, neutrophil-to-lymphocyte ratio and platelet-to-lymphocyte ratio have been described recently and can serve as important tools for PJI diagnosis.

In this review, we discuss the relevance of proven and newly described diagnostic biomarkers and provide a perspective on their practical applicability in current medical practice. In addition, we will also highlight some of the recent advances in biomarkers for the diagnosis of PJI.

## Materials and methods

The key words for literature search were total knee replacement, total hip replacement, periprosthetic joint infection, diagnostic test and biomarkers.

Original studies and meta-analyses on biomarkers in PJI between 2011 and 2022 were included for this review.

Review articles not focused on diagnostic tests, non-systematic reviews, systematic reviews without meta-analysis and articles written in languages other than English were excluded.

### Diagnosis of PJI

Early diagnosis of acute and chronic PJI is key to providing effective management and reducing the morbidity and mortality secondary to this complication. Over the past few years, the definition of PJI has been described by several organizations and societies. Of note, the International Consensus Meeting (ICM) on musculoskeletal infection first proposed its diagnostic criteria in 2013 and subsequently updated it in 2018.

As per the 2013 (ICM) criteria, it was agreed that PJI exists when [9]:

1. Two positive periprosthetic cultures with phenotypically identical organisms (Major Criteria) or
2. A sinus tract communicating with the joint (Major Criteria) or
3. Presence of 3 out of 5 of the following criteria (Minor):
  a Elevated serum C-reactive protein (CRP) AND erythrocyte sedimentation rate (ESR)
  b A single positive culture
  c Elevated synovial fluid white blood cell (WBC) count or +  + change on leukocyte esterase test strip
  d Elevated synovial fluid polymorphonuclear neutrophil percentage (PMN%)
  e Positive histological analysis of periprosthetic tissue

A New scoring-based definition for periprosthetic joint infection (PJI) is presented in Table 1 [10].

**Table 1:** New scoring-based definition (2018)

Major criteria (at least one of the following)			
Two positive cultures of the organism			Infected
Sinus tract with evidence of communication to the joint or visualization of the prosthesis			
Preoperative diagnosis	Minor criteria	Score	Decision
	Elevated CRP or D-Dimcr	2	≥6 Infected 2–5 Possibly infected^a^ 0–1 Not infected
	Elevated ESR	1	
	Elevated synovial WBC or LE	3	
	Positive alpha-defensin	3	
	Elevated synovial PMN	2	
	Elevated synovial CRP	1	
Intraoperative	Inconclusive pre-op score or dry tap	Score	Decision
	Preoperative score	-	≥6 Infected 4–5 Inconclusive^b^ ≤3 Not infected
	Positive histology	3	
	Positive purulence	3	
	Single positive culture	2	

^a^ For patients with inconclusive minor criteria, operative criteria can also be used to fulfill the definition for PJI

^b^ Consider further molecular diagnostics such as next-generation sequencing

The sensitivity and specificity of the recent diagnostic criteria are compared in the Table 2 [10].

**Table 2:** Sensitivity and specificity of various diagnostic criteria

Criteria	Sensitivity	Specificity
MSIS 2011	79.3%	99.5%
ICM 2013	86.9%	99.5%
ICM (revised) 2018	97.7%	99.5%

### Biomarkers in PJI

Biomarkers have been described by World Health Organization as “any substance, structure, or process that can be measured in the body or its products and influence or predict the incidence of outcome or disease” [11].

PJI causes activation of the innate immune system and drives white blood cells (WBCs) to produce certain substances in the serum as well as in the synovial fluid, that can be measured. Furthermore, certain specific gene expression signatures in the WBCs of the infected synovium have been exhibited using microarray techniques and these resulted in the identification of several biomarkers with diagnostic value in PJI [12].

### Serum biomarkers

#### Erythrocyte sedimentation rate (ESR) and C-reactive protein (CRP)

The ICM and AAOS (American Academy of Orthopaedic Surgeons) both recommend the use of ESR and CRP as the first line of screening in the diagnosis of PJI. These serum biomarkers have demonstrated a sensitivity of 91% and specificity of 72% for ESR and a sensitivity 94% and specificity of 74% for CRP [7, 13].

A significant drawback of both ESR and CRP is that they are non-specific markers of systemic inflammation and infection. Furthermore, the use of systemic antibiotics and immunomodulatory drugs has been shown to reduce the levels of CRP and ESR [14]. In addition to this, CRP levels are affected by immunomodulatory drugs and diagnostic cut-offs have been shown to vary with organism type. This may explain the low sensitivity of CRP in some of the recent literature.

ESR and CRP are usually elevated in the early postoperative period. ESR and CRP can be elevated for up to six weeks and 2 weeks respectively after surgery. As such, this timeline must be factored in when interpreting their values [15]. This drawback mandated that ESR and CRP should be used as minor criteria in the most recent definition, and is significant only in the presence of other minor criteria. Comparison of studies on ESR and CRP are summarised in Tables 3 and 4.

**Table 3:** Comparison of the prior studies regarding serum C-reactive protein (CRP) in the diagnosis of periprosthetic joint infection

C-reactive protein	Infection definition	Cut-off	Sensitivity	Specificity
Qin et al., JoA, 2020 [16]	MSIS 2014	7.5	81%	66%
Klim et al., Int Orthop, 2020 [17]	MSIS 2011	10.3	90%	67%
Bin et al., JoA, 2020 [18]	MSIS 2011	4.93	94%	73%
Wu et al., JoA, 2020 [19]	MSIS 2014	10.8	73%	95%
Yang et al., Sci Rep, 2021 [20]	ICM 2018	12.51	91%	83%

**Table 4:** Comparison of the prior studies regarding erythrocyte sedimentation rate in the diagnosis of periprosthetic joint infection

ESR	Infection definition	Cut-off	Sensitivity	Specificity
Qin et al., JoA, 2020 [16]	MSIS 2014	41	64%	70%
Bin et al., JoA, 2020 [18]	MSIS 2011	31	77%	97%
Wu et al., JoA, 2020 [19]	MSIS 2014	29	70%	92%
Huang et al., Orthopaedic Surgery, 2021 [21]	MSIS 2014	30	81%	88%
Yang et al., Sci Rep, 2021 [20]	ICM 2018	36.5	70%	86%

#### Procalcitonin

PCT is thought to be stimulated by bacterial lipopolysaccharides and may be a good indicator of bacterial infection [22]. A growing body of evidence has demonstrated PCT to have high specificity (98%) but have a low sensitivity (33%) for diagnosing PJI [23–25]. However, there are no data to support the application of a universal threshold for the diagnosis of PJI. Comparison of studies on Procalcitonin are summarized in Table 5.

**Table 5:** Comparison of the prior studies regarding procalcitonin in the diagnosis of PJI

Procalcitonin	Infection Definition	Cut-off (ng/mL)	Sensitivity	Specificity
Glehr et al., CORR, 2013 [25]	MSIS 2011	0.35 0.75	90% 48%	33% 100%
Randau et al., 2014 [24]	1 of the following criteria: (1) purulent synovial fluid or 1,700 leukocytes/L or 65% neutrophils in the joint aspirate (TKA) (3,600 leukocytes/L or 80% neutrophils (THA)), (2) histological confirmation of PJI, (3) pathogens detected in sterile joint aspiration or in at least two intraoperative tissue specimens, or (4) definitive signs of PJI clinically or intraoperatively (e.g., sinus tract)	0.46	13%	100%
Klim et al., Int Orthop, 2020 [17]	MSIS 2011	0.1	40%	90%

#### Interleukin-6

Interleukin 6 (IL-6) is a proinflammatory cytokine and an acute phase reactant commonly released by monocytes and macrophages. IL-6 has demonstrated promising results as a marker of inflammation following TJA and may be helpful in the diagnosis of acute PJI [15, 26]. In one study, Bottner et al. showed that IL-6 had a sensitivity of 95% and a specificity of 87% in the diagnosis of PJI [23]. In addition to this, IL-6 also demonstrated good sensitivity (80%) for PJI caused by low-virulent organisms [27].

In a recent study, Huang et al. reported that the combined sensitivity of CRP and IL-6 was 95% in the diagnosis of PJI. This demonstrates a greater potential of CRP and IL-6 detection as a screening test for PJI [28].

Serum IL-6 levels have also been shown to bear strong correlation with PJI markers, and when combined with synovial fluid WBC, demonstrated a sensitivity of 100% and a specificity of 90% [29].

However, due to variations in currently proposed cut offs, sensitivity and specificity, no recommendations are currently available for IL-6 diagnostic threshold and, as such, further studies are needed to ascertain the diagnostic value of IL-6 for PJI. Comparison of studies on Interleukin-6 are summarized in Table 6.

**Table 6:** Comparison of the prior studies regarding Interleukin 6 in the diagnosis of PJI

IL-6	Infection definition	Cut-off (pg/mL)	Sensitivity	Specificity
Bottner et al., JBJS Br, 2007 [23]	Based on findings of intraoperative culture and histology	12	95%	87%
Glehr et al., CORR, 2013 [25]	MSIS 2011	2.55	94%	53%
Ettinger et al., CID, 2015 [27]	MSIS 2011	5.12	80%	88%
Klim et al., Int Orthop, 2020 [17]	MSIS 2011	5.7	77%	70%

#### D-dimer

Apart from having utility in diagnosis of pulmonary embolism and deep vein thrombosis, a number of studies reported the role of D-dimer in diagnosis of PJI. Zhang et al., in a meta-analysis, studied 9 original research papers examining the utility of D-dimer in the diagnosis of PJI [30]. The threshold of D-dimer in 4 of the studies was 850 ng/mL and the pooled sensitivity and specificity of D-dimer for PJI diagnosis were 0.82 (95% CI, 0.72–0.89) and 0.73 (95%CI, 0.58–0.83). They concluded that D-dimer has a good diagnostic accuracy for PJI, but its specificity is not high [30]. On the other hand, a recent study found that D-dimer outperformed CRP and ESR in the diagnosis of PJI due to indolent organisms [31]. It is widely believed that D-dimer should be used with other conjunct investigations to increase the diagnostic performance of the test [30]. The studies have shown a wider variability in the threshold value, and a significant difference in results, depending on the sample type (serum or plasma). As such, thresholds for PJI should be revisited as per clinical scenario in order to improve the overall accuracy of the test [32, 33]. Comparison of studies on D-dimer are summarized in Table 7.

**Table 7:** Comparison of the prior studies regarding D-dimer in the diagnosis of periprosthetic joint infection

D-dimer	Definition	Cut-off levels	Sensitivity	Specificity
Pannu et al., JoA, 2020 [32]	ICM 2013	850	96%	32%
Qin et al., JoA, 2020 [16]	MSIS 2014	1,170	93%	75%
Wu et al., JoA, 2020 [19]	MSIS 2014	410	76%	67%
Grzelecki et al., JoR 2021 [33]	ICM 2018	850	33%	95.4%

#### Fibrinogen

Fibrinogen influences the inflammation process by activating different immune cells and by inducing the synthesis of proinflammatory cytokines, such as interleukin 6 and tumour necrosis factor [34].

Recent studies showed that fibrinogen, when used with ESR, could differentiate PJI from aseptic loosening. The optimal cut-off for fibrinogen was 3.60 g/L. This study supports the idea that fibrinogen is an adequate test to aid in the diagnosis of PJI and is not inferior to CRP in distinguishing PJI and aseptic loosening. Furthermore, it is especially useful in the assessment of infection outcomes after first-stage surgery. One study, demonstrated that fibrinogen yielded a sensitivity of 79.25% and a specificity of 94.59% in this setting [18, 35].

With the current limited literature, it is safe to say that the accuracy of fibrinogen test is not sufficient as a standalone test but should be performed in conjunction with other tests. As such, fibrinogen should be employed as an adjunct to ESR and CRP to rule out a diagnosis of PJI, but, by no means, to confirm it. Comparison of studies on fibrinogen are summarized in Table 8.

**Table 8:** Comparison of prior studies regarding fibrinogen in the diagnosis of PJI

Fibrinogen	Infection Definition	Cut-Off (mg/dL)	Sensitivity	Specificity
Wu et al., JoA, 2020 [19]	MSIS 2014	361	76%	86%
Huang et al., Orthopaedic Surgery, 2021 [21]	MSIS 2014	401	78%	88%
Yang et al., Sci Rep, 2021 [20]	ICM 2018	420	86%	90%
Huhu Wang et al., BMC Musculoskeletal Disorders [35]	MSIS 2013	382	78.48%	78.95%
Klim et al., Int Orthop, 2020 [17]	MSIS 2011	515	94%	73%
Bin et al., JoA, 2020 [18]	MSIS 2011	360	79%	95%

### Synovial fluid biomarkers

#### Leukocyte esterase (LE)

Leukocyte esterase is an enzyme secreted by activated neutrophils recruited to areas of infection.

LE strip test is a point of care test that can be performed intraoperatively while using as little as 1 mL of synovial fluid. The LE strip test was originally developed for the identification of urinary tract infections and has demonstrated its accuracy in the diagnosis of the PJI and has been subsequently integrated into the ICM criteria [13, 36, 37].

The leukocyte esterase colorimetric strip test performed by applying fluid to a reagent test strip has the advantage of being quick, easy, cheap and reliable (Chemstrip 7; Roche Diagnostics).

A potential disadvantage is the invalidation of the result by blood contamination. However, in most cases, this can be addressed by centrifugation prior to application of the fluid [38].

LE strip test has also been found to successfully differentiate between metal-on-metal failures and PJI [39].

Sensitivity and specificity of LE have been found to be 81% and 97% in a 2016 meta-analysis [40] and LE has been shown to have comparable accuracy to alpha defensin [37].

Leukocyte esterase may, however, pose some problems due to the colorimetric nature of the test. The optimal cut-off is believed to be + 2 for a high positive likelihood, and + 1 (trace) should be investigated further [41]. Even with the above limitations, as reported by a number of studies, leukocyte esterase test has an excellent diagnostic reliability in the outpatient and the operating room settings. Comparison of studies on Leukocyte esterase are summarized in Table 9.

**Table 9:** Comparison of prior studies regarding leukocyte esterase in the diagnosis of PJI

Leukocyte esterase	Infection definition	Cut-off	Sensitivity	Specificity
Parvizi et al., 2011 [42]	Own Institute^a^	+ +	80%	100%
Ruangsomboon et al., 2017 [38]	ICM Criteria	+ +	94%	87%
Guenther et al., 2014 [43]	MSIS	+ +	100%	96.5%

^a^ Own Institutional Criteria-(1) they presented with asinus tract or an open wound in communication with the joint, (2) purulence was encountered in the joint intraoperatively, (3) cultures of fluid or tissue obtained from the joint preoperatively or intraoperatively tested positive for the presence of a pathogen, or (4) elevated serum marker levels as well an elevated white blood-cell count and/or an abnormal differential cell count were observed

#### Alpha defensin

Human α-defensin is an antimicrobial peptide released by neutrophils. α-defensin has been shown to work directly and indirectly against bacterial and fungal organisms [44].

The pooled diagnostic sensitivity and specificity of α-defensin for PJI were 0.96 (95% CI, 0.87–0.99) and 0.95 (95% CI, 0.91–0.97), respectively [45].

In a recent study, the positive likelihood ratio and negative likelihood ratio of α-defensin were found to be 19.19 (95% confidence interval [CI], 9.72–37.91) and 0.05 (95% confidence interval [CI], 0.01–0.15), respectively. This finding demonstrated that a positive (or negative) result for α-defensin indicates a greatly increased (or decreased) likelihood of infection in patients undergoing revision surgery for failed hip knee or shoulder arthroplasty [45]. Both ELISA and Lateral flow technique showed a high level of accuracy but the latter demonstrated greater sensitivity [46].

The advantages of α-defensin, apart from the high accuracy, lie in that antibiotic administration and site of arthroplasty have no effect on the biomarkers while maintaining the concentration and sensitivity [47, 48].

Nevertheless, false positivity has been noted in cases of metallosis. Furthermore, although α-defensin was previously believed to achieve superior accuracy in this setting, a growing body of evidence has suggested that α-defensin provided no additional advantage when compared to conventional synovial biomarkers, such as WBC and PMN% in the diagnosis of PJI [49]. Comparison of recent studies on Alpha Defensin are summarized in Table 10.

**Table 10:** Comparison of the prior studies regarding Alpha defensin in the diagnosis of periprosthetic joint infection

Alpha defensin	Definition	Method	Sensitivity	Specificity
Sigmund et al., 2017 [47]	MSIS	Lateral Flow	69%	94%
Gehrke et al., 2018 [50]	MSIS	Lateral Flow	92.1%	100%
Riccio et al., 2018 [51]	MSIS	Lateral Flow	85%	97%
Kleiss et al., 2017 [52]	MSIS	ELISA	78%	97%
Bonanzinga et al., 2017 [53]	MSIS	ELISA	97%	97%

#### Synovial CRP and synovial white blood cell counts

Synovial CRP has been studied extensively for the diagnosis of acute and chronic PJI [54–56].

When used in conjunction with α-defensin, synovial CRP has a high specificity and sensitivity [55].

However, the varying levels in the postoperative period have made it challenging to determine a threshold for the diagnosis of acute and chronic PJI [56].

When reviewing previous studies, we noted differences in the optimal cut-off value of synovial WBC count and CRP level in diagnosing acute and chronic PJI.

Synovial WBC count and CRP level most suggestive of chronic PJI were generally believed to be more than 1,700–3,000 cells/µL and 10 mg/L, respectively [57–59].

Notably, the optimal cut off value for diagnosing acute PJI was higher than the values used for chronic PJI.

Yi et al. demonstrated that the optimal cut-off values of the synovial WBC count and CRP level were 12,800 cells/µL and 93 mg/L, respectively, within 6 weeks after primary total hip arthroplasty (THA) [60]. On the other hand, Bedair et al. reported that the optimal cut-off values of these parameters were 27,800 cells/µL and 95 mg/L, respectively, within 6 weeks after primary total knee arthroplasty (TKA) [61].

It is well-recognized that synovial CRP is a highly useful and predictive tool to diagnose infection in the acute setting (1–3 weeks after operation) and the positive and negative predictive values are significantly higher when used in combination with synovial WBC counts [56].

#### Interleukin IL-1β

IL-1β is a multifunctional and highly potent pro-inflammatory cytokine that is associated with bone resorption in some inflammatory diseases [62]. Nicolas et al. found that IL-1β played an important role in early control of the bacterial burden in patients who had undergone primary TJA [63]. This biomarker has since shown excellent utility in the diagnosis of PJI.

The threshold used in a recent 2021 study was 312.7 pg/mL for diagnosing chronic PJI [64]. IL-1β is a biomarker which is rarely used, but it may have utility in differentiating aseptic loosening and chronic PJI.

### Novel biomarkers

#### Soluble Pe Cam-1

This is an immunologically reactive molecule that is shed from the surface of native T-cells upon activation. Soluble Pe Cam-1 has been described to be significantly higher in individuals with septic shock.

A novel study found the specificity to be 80% and sensitivity 82%, when using a threshold value of 54.3 ng Soluble Pe Cam-1/mL synovial fluid to define an infectious status [65].

The need for immediate testing and storage at -80° for delayed testing is reportedly a drawback [65].

This modality might have a role in the future in excluding PJI in patients with aseptic loosening.

#### Lipocalcin

Neutrophilic gelatinase-associated lipocalin (NGAL) is bacteriostatic and secreted during bacterial infections by hepatocytes, renal tubular cells, and immune cells (neutrophils and macrophages in particular) [66, 67]. White blood cell (WBC) count is an established and reliable marker for PJI and lipocalcin is produced by the most abundant WBC.

Deirmeingian et al. were the first to examine the role of this marker in the diagnosis of PJI and demonstrated a sensitivity and a specificity of 100% when using the MSIS criteria as the gold standard [68]. Subsequently, Vergara et al. found that lipocalcin had a sensitivity of 86% and a specificity of 77% in the diagnosis of PJI [69]. However, a universal diagnostic threshold for lipocalcin has yet to be determined. In limited reports, the generalized WBC count has been found to be more accurate than the more specific lipocalcin [70].

#### Calprotectin

Calprotectin is an important pro-inflammatory factor of the innate immune system that acts as an endogenous damage-associated molecular pattern molecule via toll-like receptor 4 activation [71].

The calprotectin synovial fluid test has demonstrated a sensitivity of 100% (95% CI, 99.96–100) and a specificity of 95% (95% CI, 89.4–100) for diagnosing PJI [72].

Many authors consider calprotectin to be a more sensitive marker of disease activity in patients with rheumatoid disorders, when compared to conventional inflammatory markers such as the ESR and CRP [73].

The concentration of synovial calprotectin potentially reflects the number and activity of white blood cells in a localized compartment [72]. ELISA has been used in the two studies and has shown promising results. It has the potential to be used as a rule-out test [74].

Additional work including multicentre studies is needed to evaluate the performance of synovial calprotectin test and to define its accuracy and its role in the diagnosis of infected arthroplasty.

#### Synovial fluid D-lactate

More recently, D-lactate has been proposed as a new point of care bio-marker. It is a substance produced by the bacteria and may be the future of synovial fluid investigation [75, 76]. However, due to the high false positivity rate of this test and variable cut-offs, depending on the virulence of the organism, more research is needed prior to widespread adoption [77].

#### Monocyte-to-lymphocyte ratio (MLR), neutrophil-to-lymphocyte ratio (NLR)

The neutrophil-to-lymphocyte ratio and monocyte-to-neutrophil ratio have been found useful in differentiating between bacterial and viral infections and predicting outcomes [78]. There were limited studies relating NLR and MLR to PJI. Jiang et al. looked at the association between NLR or MLR and PJI in septic arthritis patients undergoing THA to detect any occult infections and suggested that NLR and MLR are unreliable biomarkers [79]. In a preliminary study, Zhao et al. observed higher values of NLR and MLR during the incubation period in the cases of PJI, and suggested that it is potentially useful in early postoperative infection [80]. These biomarkers, in combination with plasma fibrinogen, ESR and CRP have been shown to increase the diagnostic performance [81]. Although a recent meta-analysis on the aforementioned ratios has shown a fair diagnostic value, it is not suggested as a screening tool [82]. More studies are needed to reach a consensus on these biomarkers.

#### Platelet-to-lymphocyte ratio(PLR), Platelet-to-mean platelet Volume Ratio (PVR)

Blood platelets have been known for their roles in mediating the body’s innate response to both acute and chronic inflammation. Van der Lelie et al. reported an increase in mean platelet volume (MPV) due to septicemia [83]. Many recent articles reported PLR and PVR as markers for systemic inflammation [84–86], and proved them to be an inexpensive alternative for PJI detection.

Paziuk et al. demonstrated the association between PVR and PJI, and reported better outcomes when using PVR in conjunction with ESR and CRP in predicting PJI, rather than ESR or CRP alone [87]. These results were corroborated by Tirumala et al. who observed the highest sensitivity of 87.7% for PVR at the optimal threshold of 30.82, and a specificity of 82.5% at a threshold of 234.13 for PLR. They also suggest that both PLR and PVR, when used with Musculoskeletal Infection Society thresholds for serum biomarkers (ESR, CRP) and synovial biomarkers (WBC and PMN%), can achieve significantly higher sensitivity and specificity rates for PJI at or above 97%. It has been suggested that the combined diagnostic value outperforms the combination of WBC and PMN% (aspirate samples) in terms of sensitivity and specificity [88]. These biomarkers are promising and require more studies for further validation.

## Conclusion

Accurate preoperative diagnosis of PJI presents a challenge for clinicians, especially in the cases of low-virulence organisms.

The serum parameters, in general, have insufficient accuracy for diagnosing PJI due to false positivity in patients with systemic inflammatory disorders, and autoimmune diseases. Lower sensitivities can be explained by improper immune response in patients with encapsulated joint infection (forming bio-films) and those being administrated systemic antibiotics.

Currently, serum CRP, IL-6 and fibrinogen seem to perform best in terms of accuracy among the presented biomarkers. Although the accuracy is limited, we recommend these three parameters in the preoperative diagnosis of PJI as suggestive criteria. However, results should be interpreted with caution in the clinical practice. On the other hand, synovial biomarkers are much more specific and sensitive than their serum counterparts. Alpha defensin and leukocyte esterase have proven their accuracy in multiple studies in determining presence of infection. In our experience, CRP, ESR, synovial CRP are all good predictors of PJI. Alpha-defensin and leukocyte esterase are also useful diagnostic test with good applicability.

## Data Availability

They are available from (1) Pubmed, (2) Medline AND (3) Indian joint registry.

## Abbreviations

ALP: Aseptic loosening of prosthesis
PJI: Periprosthetic joint infection
ECDC: European Centre for Disease Prevention and Control’s
SSI: Surgical site infection
TJA: Total joint arthroplasty
ICM: International consensus meeting
CRP: C-reactive protein
ESR: Erythrocyte sedimentation rate
WBC: White blood cell
PCT: Procalcitonin
IL-6: Interleukin 6
LE: Leukocyte esterase
ELISA: Enzyme-Linked Immunosorbent Assay
THA: Total hip arthroplasty
TKA: Total knee arthroplasty
IL: Interleukin
NGAL: Neutrophilic gelatinase- associated lipocalin
MLR: Monocyte-Lymphocyte ratio
NLR: Neutrophil to lymphocyte ratio
PLR: Platelet to Lymphocyte ratio
PVR: Platelet to Mean platelet Volume Ratio

## References

[1] Brooks PM (2006). The burden of musculoskeletal disease–a global perspective. Clin Rheumatol.

[2] Nugent R (2008). Chronic diseases in developing countries: health and economic burdens. Ann N Y Acad Sci.

[3] European Center for Disease Prevention and Control. Healthcare-associated infections: surgical site infections. In: Annual epidemiological report for 2017. Stockholm: Ecdc; 2019.

[4] Tande AJ, Patel R (2014). Prosthetic joint infection. Clin Microbiol Rev.

[5] Sharkey PF, Lichstein PM, Shen C, Tokarski AT, Parvizi J (2014). Why are total knee arthroplasties failing today–has anything changed after 10 years?. J Arthroplasty.

[6] Kamath AF, Ong KL, Lau E, Chan V, Vail TP, Rubash HE (2015). Quantifying the burden of revision total joint arthroplasty for periprosthetic infection. J Arthroplasty.

[7] Parvizi J, Adeli B, Zmistowski B, Restrepo C, Greenwald AS (2012). Management of periprosthetic joint infection: the current knowledge - AAOS exhibit selection. J Bone Joint Surg.

[8] Vaidya SV, Jogani AD, Pachore JA, Armstrong R, Vaidya CS (2021). India joining the world of hip and knee registries: present status—a leap forward. Indian J Orthop.

[9] Parvizi J, Gehrke T (2014). Definition of periprosthetic joint infection. J Arthroplasty.

[10] Parvizi J, Tan TL, Goswami K, Higuera C, Della Valle C, Chen AF (2018). The 2018 definition of periprosthetic hip and knee infection: an evidence-based and validated criteria. J Arthroplasty.

[11] Organization WH, on Chemical Safety IP. Biomarkers in risk assessment : validity and validation. Geneva: World Health Organization; 2001. p. Summary and conclusions in French (p. 235–236) and. (Environmental health criteria ; 222).

[12] Deirmengian C, Lonner JH, Booth REJ (2005). The Mark Coventry award: white blood cell gene expression: a new approach toward the study and diagnosis of infection. Clin Orthop Relat Res.

[13] Parvizi J, Gehrke T, Chen AF (2013). Proceedings of the international consensus on periprosthetic joint infection. Bone Jt J.

[14] Shahi A, Deirmengian C, Higuera C, Chen A, Restrepo C, Zmistowski B (2015). Premature therapeutic antimicrobial treatments can compromise the diagnosis of late periprosthetic joint infection. Clin Orthop Relat Res.

[15] Shahi AS, Parvizi J (2016). The role of biomarkers in the diagnosis of periprosthetic joint infection. EFORT Open Rev.

[16] Qin L, Li F, Gong X, Wang J, Huang W, Hu N. Combined Measurement of D-Dimer and C-Reactive Protein Levels: Highly Accurate for Diagnosing Chronic Periprosthetic Joint Infection. J Arthroplasty [Internet]. 2020;35(1):229–34. Available from: 10.1016/j.arth.2019.08.012. 10.1016/j.arth.2019.08.012 31526698

[17] Klim SM, Amerstorfer F, Glehr G, Hauer G, Smolle MA, Leitner L (2020). Combined serum biomarker analysis shows no benefit in the diagnosis of periprosthetic joint infection. Int Orthop.

[18] Bin G, Xinxin Y, Fan L, Shenghong W, Yayi X (2020). Serum fibrinogen test performs well for the diagnosis of periprosthetic joint infection. J Arthroplasty.

[19] Wu H, Meng Z, Pan L, Liu H, Yang X, Yongping C (2020). Plasma fibrinogen performs better than plasma D-dimer and fibrin degradation product in the diagnosis of periprosthetic joint infection and determination of reimplantation timing. J Arthroplasty.

[20] Yang F, Zhao C, Huang R, Ma H, Wang X, Wang G (2021). Plasma fibrinogen in the diagnosis of periprosthetic joint infection. Sci Rep.

[21] Huang J-C, Chen X, Qiang S, Zheng W, Zheng J, Jin Y (2021). Exciting performance of plasma fibrinogen in periprosthetic joint infection diagnosis. Orthop Surg.

[22] Vijayan AL, Ravindran S, Saikant R, Lakshmi S, Kartik R, Manoj G (2017). Procalcitonin: a promising diagnostic marker for sepsis and antibiotic therapy. J Intensive Care.

[23] Bottner F, Wegner A, Winkelmann W, Becker K, Erren M, Götze C (2007). Interleukin-6, procalcitonin and TNF-α. J Bone Jt Surg Ser B.

[24] Randau TM, Friedrich MJ, Wimmer MD, Reichert B, Kuberra D, Stoffel-Wagner B (2014). Interleukin-6 in serum and in synovial fluid enhances the differentiation between periprosthetic joint infection and aseptic loosening. PLoS ONE.

[25] Glehr M, Friesenbichler J, Hofmann G, Bernhardt GA, Zacherl M, Avian A (2013). Novel biomarkers to detect infection in revision hip and knee arthroplasties infection. Clin Orthop Relat Res.

[26] Wirtz DC, Heller KD, Miltner O, Zilkens KW, Wolff JM (2000). Interleukin-6: a potential inflammatory marker after total joint replacement. Int Orthop.

[27] Ettinger M, Calliess T, Kielstein JT, Sibai J, Brückner T, Lichtinghagen R (2015). Circulating biomarkers for discrimination between aseptic joint failure, low-grade infection, and high-grade septic failure. Clin Infect Dis an Off Publ Infect Dis Soc Am.

[28] Huang ZY, Huang Q, Wang LY, Lei YT, Xu H, Shen B (2020). Normal trajectory of Interleukin-6 and C-reactive protein in the perioperative period of total knee arthroplasty under an enhanced recovery after surgery scenario. BMC Musculoskelet Disord.

[29] Majors I, Jagadale VS (2019). Serum interleukin 6 could be a valuable initial diagnostic tool in prosthetic knee joint infections. Eur J Orthop Surg Traumatol.

[30] Zhang H, Sun X, Xin P, Zhu X, Jie K, Cao H (2020). Diagnostic accuracy of D-dimer in periprosthetic joint infection: a diagnostic meta-analysis. J Orthop Surg Res.

[31] Tarabichi S, Goh GS, Baker CM, Chisari E, Shahi A, Parvizi J (2023). Plasma D-dimer is noninferior to serum C-reactive protein in the diagnosis of periprosthetic joint infection. J Bone Joint Surg Am.

[32] Pannu TS, Villa JM, Patel PD, Riesgo AM, Barsoum WK, Higuera CA (2020). The utility of serum D-dimer for the diagnosis of periprosthetic joint infection in revision total hip and knee arthroplasty. J Arthroplasty.

[33] Grzelecki D, Walczak P, Grajek A, Szostek M, Dudek P, Bartosz P (2021). Elevated plasma D-dimer concentration has higher efficacy for the diagnosis of periprosthetic joint infection of the knee than of the hip—a single-center, retrospective study. J Orthop Res.

[34] Jensen T, Kierulf P, Sandset PM, Klingenberg O, Joø GB, Godal HC (2007). Fibrinogen and fibrin induce synthesis of proinflammatory cytokines from isolated peripheral blood mononuclear cells. Thromb Haemost.

[35] Wang H, Zhou H, Jiang R, Qian Z, Wang F, Cao L (2021). Globulin, the albumin-to-globulin ratio, and fibrinogen perform well in the diagnosis of periprosthetic joint infection. BMC Musculoskelet Disord.

[36] Leighton PM, Little JA (1985). Leucocyte esterase determination as a secondary procedure for urine screening. J Clin Pathol.

[37] Yu BZ, Li R, Fu J, Chai W, Hao LB, Chen JY (2021). Leukocyte esterase test and alpha-defensin test have similar accuracy for the diagnosis of periprosthetic joint infection. Int Orthop.

[38] Ruangsomboon P, Chinprasertsuk S, Khejonnit V, Chareancholvanich K (2017). Effect of depth of centrifuged synovial fluid on leukocyte esterase test for periprosthetic joint infection. J Orthop Res.

[39] Tischler EH, Plummer DR, Chen AF, Della Valle CJ, Parvizi J (2016). Leukocyte esterase: metal-on-metal failure and periprosthetic joint infection. J Arthroplasty.

[40] Wyatt MC, Beswick AD, Kunutsor SK, Wilson MJ, Whitehouse MR, Blom AW (2016). The alpha-defensin immunoassay and leukocyte esterase colorimetric strip test for the diagnosis of periprosthetic infection. J Bone Jt Surg Am.

[41] Tarabichi M, Fleischman AN, Shahi A, Tian S, Parvizi J (2017). Interpretation of leukocyte esterase for the detection of periprosthetic joint infection based on serologic markers. J Arthroplasty.

[42] Parvizi J, Jacovides C, Antoci V, Ghanem E. Diagnosis of periprosthetic joint infection: the utility of a simple yet unappreciated enzyme. J Bone Joint Surg Am. 2011;93(24):2242–8. 10.2106/JBJS.J.01413 22258769

[43] Guenther D, Kokenge T, Jacobs O, Omar M, Krettek C, Gehrke T (2014). Excluding infections in arthroplasty using leucocyte esterase test. Int Orthop..

[44] Ganz T, Selsted ME, Szklarek D, Harwig SS, Daher K, Bainton DF (1985). Defensins. Natural peptide antibiotics of human neutrophils. J Clin Invest.

[45] Yuan J, Yan Y, Zhang J, Wang B, Feng J (2017). Diagnostic accuracy of alpha-defensin in periprosthetic joint infection: a systematic review and meta-analysis. Int Orthop.

[46] Kuiper JWP, Verberne SJ, Vos SJ, van Egmond PW (2020). Does the alpha defensin ELISA test perform better than the alpha defensin lateral flow test for PJI diagnosis? A systematic review and meta-analysis of prospective studies. Clin Orthop Relat Res.

[47] Sigmund IK, Holinka J, Gamper J, Staats K, Böhler C, Kubista B (2017). Qualitative ?-defensin test (Synovasure) for the diagnosis of periprosthetic infection in revision total joint arthroplasty. Bone Jt J.

[48] Frangiamore SJ, Saleh A, Grosso MJ, Kovac MF, Higuera CA, Iannotti JP (2015). α-Defensin as a predictor of periprosthetic shoulder infection. J Shoulder Elb Surg.

[49] Ivy MI, Sharma K, Greenwood-Quaintance KE, Tande AJ, Osmon DR, Berbari EF (2021). Synovial fluid α defensin has comparable accuracy to synovial fluid white blood cell count and polymorphonuclear percentage for periprosthetic joint infection diagnosis. Bone Jt J.

[50] Gehrke T, Lausmann C, Citak M, Bonanzinga T, Frommelt L, Zahar A. The accuracy of the alpha defensin lateral flow device for diagnosis of periprosthetic joint infection. J Bone Jt Surg - Am Vol. 2018;100(1):42–8. 10.2106/JBJS.16.01522 29298259

[51] Riccio G, Cavagnaro L, Akkouche W, Carrega G, Felli L, Burastero G. Qualitative Alpha-defensin Versus The Main Available Tests For The Diagnosis Of Periprosthetic Joint Infection: Best Predictor Test? J bone Jt Infect. 2018;3(3):156–64. 10.7150/jbji.26401 PMC6098818 30128266

[52] Kleiss S, Jandl NM, Novo de Oliveira A, Rüther W, Niemeier A. Diagnostic accuracy of alpha-defensin enzyme-linked immunosorbent assay in the clinical evaluation of painful hip and knee arthroplasty with possible prosthetic joint infection: a prospective study of 202 cases. Bone Joint J. 2019;101-B(8):970–7. 10.1302/0301-620X.101B8.BJJ-2018-1390.R2 31362542

[53] Bonanzinga T, Zahar A, Dütsch M, Lausmann C, Kendoff D, Gehrke T. How Reliable Is the Alpha-defensin Immunoassay Test for Diagnosing Periprosthetic Joint Infection? A Prospective Study. Clin Orthop Relat Res. 2017;475(2):408–15. 10.1007/s11999-016-4906-0 PMC5213924 27343056

[54] Omar M, Ettinger M, Reichling M, Petri M, Guenther D, Gehrke T (2015). Synovial C-reactive protein as a marker for chronic periprosthetic infection in total hip arthroplasty. Bone Jt J.

[55] Stone WZ, Gray CF, Parvataneni HK, Al-Rashid M, Vlasak RG, Horodyski MB (2018). Clinical evaluation of synovial alpha defensin and synovial C-reactive protein in the diagnosis of periprosthetic joint infection. J Bone Jt Surg Am.

[56] Kim SG, Kim JG, Jang KM, Han SB, Lim HC, Bae JH (2017). Diagnostic value of synovial white blood cell count and serum C-reactive protein for acute periprosthetic joint infection after knee arthroplasty. J Arthroplasty.

[57] Della Valle CJ, Sporer SM, Jacobs JJ, Berger RA, Rosenberg AG, Paprosky WG (2007). Preoperative testing for sepsis before revision total knee arthroplasty. J Arthroplasty.

[58] Trampuz A, Hanssen AD, Osmon DR, Mandrekar J, Steckelberg JM, Patel R (2004). Synovial fluid leukocyte count and differential for the diagnosis of prosthetic knee infection. Am J Med.

[59] Parvizi J, Ghanem E, Menashe S, Barrack RL, Bauer TW (2006). Periprosthetic infection: what are the diagnostic challenges?. J Bone Joint Surg Am.

[60] Yi PH, Cross MB, Moric M, Sporer SM, Berger RA, Della Valle CJ (2014). The 2013 Frank Stinchfield award: diagnosis of infection in the early postoperative period after total hip arthroplasty. Clin Orthop Relat Res.

[61] Bedair H, Ting N, Jacovides C, Saxena A, Moric M, Parvizi J (2011). The Mark Coventry award: diagnosis of early postoperative TKA infection using synovial fluid analysis. Clin Orthop Relat Res.

[62] Lorenzo J, Horowitz M, Choi Y (2008). Osteoimmunology: interactions of the bone and immune system. Endocr Rev.

[63] Bernthal NM, Pribaz JR, Stavrakis AI, Billi F, Cho JS, Ramos RI (2011). Protective role of IL-1β against post-arthroplasty staphylococcus aureus infection. J Orthop Res Off Publ Orthop Res Soc.

[64] Wang H, Qin L, Wang J, Huang W (2021). Synovial fluid IL-1β appears useful for the diagnosis of chronic periprosthetic joint infection. J Orthop Surg Res.

[65] Fuchs M, Trampuz A, Kirschbaum S, Winkler T, Andrea SF (2021). Soluble pecam-1 as a biomarker in periprosthetic joint infection. J Clin Med.

[66] Schmidt-Ott KM, Mori K, Jau YL, Kalandadze A, Cohen DJ, Devarajan P (2007). Dual action of neutrophil gelatinase-associated lipocalin. J Am Soc Nephrol.

[67] Nairz M, Haschka D, Demetz E, Weiss G (2014). Iron at the interface of immunity and infection. Front Pharmacol.

[68] Deirmengian C, Kardos K, Kilmartin P, Cameron A, Schiller K, Parvizi J (2014). Diagnosing periprosthetic joint infection: has the era of the biomarker arrived?. Clin Orthop Relat Res.

[69] Vergara A, Fernández-Pittol MJ, Muñoz-Mahamud E, Morata L, Bosch J, Vila J (2019). Evaluation of lipocalin-2 as a biomarker of periprosthetic joint infection. J Arthroplasty.

[70] Dijkman C, Thomas AR, Koenraadt KLM, Ermens AAM, van Geenen RCI (2020). Synovial neutrophilic gelatinase-associated lipocalin in the diagnosis of periprosthetic joint infection after total knee arthroplasty. Arch Orthop Trauma Surg.

[71] Vogl T, Tenbrock K, Ludwig S, Leukert N, Ehrhardt C, van Zoelen MAD (2007). Mrp8 and Mrp14 are endogenous activators of Toll-like receptor 4, promoting lethal, endotoxin-induced shock. Nat Med.

[72] Salari P, Grassi M, Cinti B, Onori N, Gigante A (2020). Synovial fluid calprotectin for the preoperative diagnosis of chronic periprosthetic joint infection. J Arthroplasty.

[73] Abildtrup M, Kingsley G, Scott D (2015). Calprotectin as a biomarker for rheumatoid arthritis: a systematic review. J Rheumatol.

[74] Warren J, Anis HK, Bowers K, Pannu T, Villa J, Klika AK (2021). Diagnostic utility of a novel point-of-care test of calprotectin for periprosthetic joint infection after total knee arthroplasty: a prospective cohort study. J Bone Joint Surg Am.

[75] Marcos MA, Vila J, Gratacos J, Brancos MA, Jimenez de Anta MT (1991). Determination of D-lactate concentration for rapid diagnosis of bacterial infections of body fluids. Eur J Clin Microbiol Infect Dis Off Publ Eur Soc Clin Microbiol.

[76] Yermak K, Karbysheva S, Perka C, Trampuz A, Renz N (2019). Performance of synovial fluid D-lactate for the diagnosis of periprosthetic joint infection: a prospective observational study. J Infect.

[77] Karbysheva S, Yermak K, Grigoricheva L, Renz N, Perka C, Trampuz A (2020). Synovial fluid d-lactate-a novel pathogen-specific biomarker for the diagnosis of periprosthetic joint infection. J Arthroplasty.

[78] Naess A, Nilssen SS, Mo R, Eide GE, Sjursen H (2017). Role of neutrophil to lymphocyte and monocyte to lymphocyte ratios in the diagnosis of bacterial infection in patients with fever. Infection.

[79] Jiang W, Xu H, Wang X, Xie J, Huang Q, Zhou Z (2022). Poor performance of monocyte-to-lymphocyte ratio, neutrophil-to-lymphocyte ratio, and fibrinogen when screening for occult infection among patients with sequelae of suppurative hip arthritis before total hip arthroplasty. Int Orthop.

[80] Zhao G, Chen J, Wang J, Wang S, Xia J, Wei Y (2020). Predictive values of the postoperative neutrophil-to-lymphocyte ratio, platelet-to-lymphocyte ratio, and lymphocyte-to-monocyte ratio for the diagnosis of early periprosthetic joint infections: a preliminary study. J Orthop Surg Res.

[81] Maimaiti Z, Xu C, Fu J, Chai W, Zhou Y, Chen J (2022). The potential value of monocyte to lymphocyte ratio, platelet to mean platelet volume ratio in the diagnosis of periprosthetic joint infections. Orthop Surg.

[82] Festa E, Ascione T, Bernasconi A, Di Gennaro D, Basso MA, Guarino A (2022). Diagnostic performance of neutrophil to lymphocyte ratio, monocyte to lymphocyte ratio, platelet to lymphocyte ratio, and platelet to mean platelet volume ratio in periprosthetic hip and knee infections: a systematic review and meta-analysis. Diagnostics (Basel.).

[83] Van der Lelie J, Von dem Borne AK (1983). Increased mean platelet volume in septicaemia. J Clin Pathol.

[84] Oh GH, Chung SP, Park YS, Hong JH, Lee HS, Chung HS (2017). Mean platelet volume to platelet count ratio as a promising predictor of early mortality in severe sepsis. Shock.

[85] Erre GL, Paliogiannis P, Castagna F, Mangoni AA, Carru C, Passiu G (2019). Meta-analysis of neutrophil-to-lymphocyte and platelet-to-lymphocyte ratio in rheumatoid arthritis. Eur J Clin Invest.

[86] Gasparyan AY, Ayvazyan L, Mukanova U, Yessirkepov M, Kitas GD (2019). The platelet-to-lymphocyte ratio as an inflammatory marker in rheumatic diseases. Ann Lab Med.

[87] Paziuk T, Rondon AJ, Goswami K, Tan TL, Parvizi J (2020). A novel adjunct indicator of periprosthetic joint infection: platelet count and mean platelet volume. J Arthroplasty.

[88] Tirumala V, Klemt C, Xiong L, Chen W, van den Kieboom J, Kwon YM (2021). Diagnostic utility of platelet count/lymphocyte count ratio and platelet count/mean platelet volume ratio in periprosthetic joint infection following total knee arthroplasty. J Arthroplasty.

